# High-Quality Draft Genome Sequence of the Causal Agent of the Current Panama Disease Epidemic

**DOI:** 10.1128/MRA.00904-19

**Published:** 2019-09-05

**Authors:** Rachel J. Warmington, William Kay, Aaron Jeffries, Paul O’Neill, Audrey Farbos, Karen Moore, Daniel P. Bebber, David J. Studholme

**Affiliations:** a Eden Project, Bodelva, United Kingdom; b Department of Biosciences, University of Exeter, Exeter, United Kingdom; Vanderbilt University

## Abstract

We present a high-quality draft genome assembly for Fusarium oxysporum f. sp. *cubense* tropical race 4 (Fusarium odoratissimum), assembled from PacBio reads and consisting of 15 contigs with a total assembly size of 48.59 Mb. This strain appears to belong to vegetative compatibility group complex 01213/16.

## ANNOUNCEMENT

Ascomycete fungus Fusarium oxysporum f. sp. *cubense* is the causal agent of Fusarium wilt (Panama disease), which decimated banana plantations in the 1950s (1–3). The tropical race 4 (TR4) variant of F. oxysporum f. sp. *cubense*, also known as Fusarium odoratissimum (4), is responsible for a current epidemic spreading through Asia, Africa, and Latin America (4–9). Here, we present a high-quality draft sequence of F. oxysporum TR4; previously available TR4 genome assemblies were assembled from short reads (10) and were highly fragmented. The data from a recently reported PacBio-based TR4 assembly are not publicly available (11).

We isolated F. oxysporum f. sp. *cubense* TR4 strain UK0001 from a symptomatic banana plant (Musa sp.) at the Eden Project (Cornwall, UK). Genomic DNA was extracted from microconidia (that is, asexual spores) cultured in potato dextrose broth, shaken at 140 rpm, and stored at 25°C for 5 days. Lysis buffer (500 μl; 1% SDS, 100 mM Tris, 10 mM EDTA) was added to a pellet of filtered and washed spores before it was placed on a Vibrax for 30 mins at 2,500 rpm. Five hundred microliters of 25:24:1 phenol-chloroform-isoamyl alcohol was added to tubes before vortexing for 60 s and centrifuging for 10 min at 16,000 × *g*. The resulting supernatant was mixed with 28 μl cold 7.5 M ammonium acetate and 204 μl cold isopropanol and incubated at −20°C overnight. The following day, the pellets were washed twice in 70% ethanol and twice in 96% ethanol before tubes were inverted and air dried for 60 min at room temperature. Pellets were resuspended in 40 μl MilliQ water and treated with RNase I_f_ (Biolabs, UK).

The genome was sequenced using one PacBio single-molecule real-time (SMRT) cell with v3.0 chemistry. Libraries were prepared with the SMRTbell Express template preparation kit and size selected with a 15-kb cutoff using BluePippin (Sage Science, MA, USA). Additionally, we used the Illumina MiSeq system to sequence paired-end libraries prepared with the NEXTFLEX 8-barcode kit (Perkin Elmer), generating 2,889,905 pairs of 300-bp reads.

A total of 723,327 filtered PacBio subreads (*N*_50_ length, 19,851 bp) were assembled using the Hierarchical Genome Assembly Process (HGAP) v4 in SMRT Link v7.0.0.63985 (12). Illumina sequence reads were not used in the assembly. The assembly yielded 15 contigs with a total assembly size of 48,588,396 bp (47.54% G+C content, *N*_50_ length of 4,494,293 bp). Completeness was estimated using the Sordariomycetes data set in Benchmarking Universal Single-Copy Orthologs (BUSCO) v3.0.2 (13, 14). Of 3,725 target genes, 3,676 (98.6%) occurred as intact and single copies, similar to the 3,673 in a recent PacBio-based assembly of an F. oxysporum f. sp. *cubense* race 1 genome (15).

We used the MAKER pipeline (16, 17) v2.31.10, including AUGUSTUS (18) v3.1 for *ab initio* gene prediction with the “*Fusarium*” species option and homology evidence from a set of 53,031 F. oxysporum f. sp. *cubense* proteins from the NCBI Proteins database (19) after soft masking by RepeatMasker v4.0.7 against the “Ascomycota” section of RepBase (20–22). This predicted 14,472 protein-coding genes. BLASTN searches against discriminative sequences (23) suggest that UK0001 belongs to vegetative compatibility group complex 01213/16.

### Data availability.

These data are deposited at DDBJ/ENA/GenBank under the accession number VMNF00000000 and the Sequence Read Archive (24) under BioProject accession number PRJNA556111.
